# Droplets as Carriers for Flexible Electronic Devices

**DOI:** 10.1002/advs.201901862

**Published:** 2019-10-30

**Authors:** Mingxing Zhou, Ziyue Wu, Yicong Zhao, Qing Yang, Wei Ling, Ya Li, Hang Xu, Cheng Wang, Xian Huang

**Affiliations:** ^1^ Department of Biomedical Engineering Tianjin University 92 Weijin Road Tianjin 300072 P. R. China; ^2^ Department of Mechanical Engineering Missouri University of Science and Technology 400 West 13th Street Rolla MO 65401 USA

**Keywords:** active droplets, controllable motions, environmental adaptability, flexible electronics, multifunctionality

## Abstract

Coupling soft bodies and dynamic motions with multifunctional flexible electronics is challenging, but is essential in satisfying the urgent and soaring demands of fully soft and comprehensive robotic systems that can perform tasks in spite of rigorous spatial constraints. Here, the mobility and adaptability of liquid droplets with the functionality of flexible electronics, and techniques to use droplets as carriers for flexible devices are combined. The resulting active droplets (ADs) with volumes ranging from 150 to 600 µL can conduct programmable functions, such as sensing, actuation, and energy harvesting defined by the carried flexible devices and move under the excitation of gravitational force or magnetic force. They work in both dry and wet environments, and adapt to the surrounding environment through reversible shape shifting. These ADs can achieve controllable motions at a maximum velocity of 226 cm min^−1^ on a dry surface and 32 cm min^−1^ in a liquid environment. The conceptual system may eventually lead to individually addressable ADs that offer sophisticated functions for high‐throughput molecule analysis, drug assessment, chemical synthesis, and information collection.

## Graphical abstract

Droplets as carriers for flexible electronics are presented. Active droplets can adapt themselves to sounding environment and conduct programmable functions defined by the carried flexible devices. The concept that combines liquid droplets with flexible electronics aims to promote individually addressable active droplets that offer sophisticated functions for high‐throughput molecule analysis, drug assessment, chemical synthesis, and information collection.

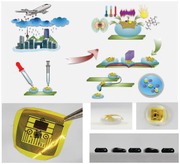

## 1 Introduction

Flexible electronic^1^, ^2^, ^3^, ^4^, ^5^ devices are promising replacement for current rigid circuits in electronics that demands integration with soft tissues and curvilinear surface. They possess similar mechanics as soft organs and supply sophisticated functions that have been demonstrated for sensing, displaying, computing, and energy harvesting and storage. The mobility of flexible devices depends highly on their hosting objects, which typically involve organisms, machines, or electronics that are fixed on locations. Inspired by biological organisms that possess soft mechanics and comply readily with external forces, soft robots that can withstand large deformation through reversible shape‐shifting have been demonstrated with functions such as motion,^6^, ^7^, ^8^ grasping,^9^ transportation,^10^ and tactile sensing.^11^, ^12^ However, functionalization of existing soft robots still relies primarily on rigid components and circuits, urging the need to realize fully soft robots that offer comprehensive functions based completely on soft matters. The combination of flexible electronics with soft robots may improve mobility of flexible electronics, allowing flexible devices to perform tasks on different locations and environments.

Liquid droplets have extreme softness and excellent compliance to surrounding environment. They are widely available in nature and easily controllable by optical,^13^ electrical,^14^ and chemical^15^ actuation approaches. They may serve as an ideal platform to host flexible electronics devices and may lead to a new type of soft robots that differ from current elastomer‐based systems. Despite many research in integrating flexible electronic devices onto different objects with varied surface properties, their integration with droplets have not yet been explored. Challenging issues such as device deployment on droplets, liquid/device interaction, device actuation, and potential applications have not yet been resolved.

Here, we present techniques to integrate flexible electronics with droplets, resulting in active droplets (ADs) that maintain their integrity purely by surface tension without the need for physical shells. The ADs possess volumes ranging from 150 to 600 µL, and perform customized functions such as sensing, actuation, and energy harvesting defined by the carried flexible devices. The ADs offer free and reversible droplet deformation in response to surrounding environments, and achieve a maximum velocity of 226 cm min^−1^ on a dry surface and 32 cm min^−1^ in a liquid environment actuated through magnetic forces. To our best knowledge, this is the first investigation that combines flexible electronics with liquid droplets. This work may eventually lead to intellective hybrid electronic systems enabled by individually addressable ADs with autonomous perception and control capabilities, allowing potential applications in environmental monitoring, molecule analysis, drug assessment, and chemical synthesis.

## 2 Results

### 2.1 Concept and Design of Active Droplets

**Figure 1:**
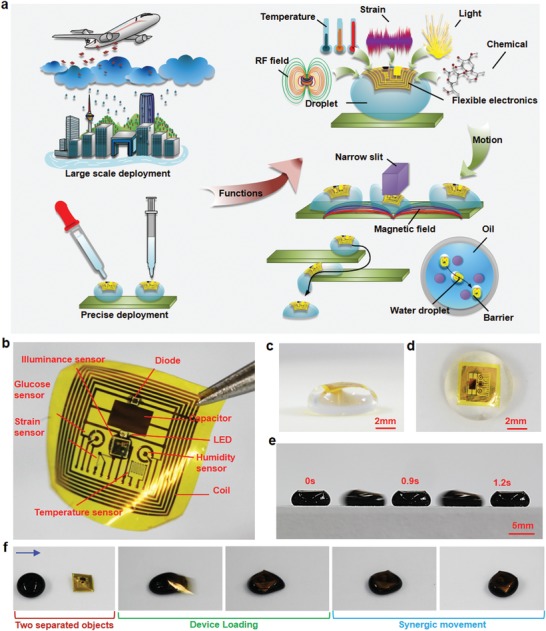
Schematics of active droplets (ADs). a) A schematics of the ADs that can be deployed through large‐scale precipitation and precise dispensing. The droplets can integrate with different functions determined by the carrying flexible electronics devices, and move together with the device to complete complicated motions in dry and wet environments. b) An image of an as‐fabricated flexible device clamped on the edge by a tweezer. Images of c) side view and d) top view of an AD. e) A time‐series image of dynamic motion of a magnetofluidic (MF) AD. f) A flexible electronics device deployment with an MF AD

The fundamental concept of ADs is demonstrated in **Figure** **1**a The ADs can be deployed in large quantities through precipitation, resulting in descent of flexible electronic devices with rainfall. The flexible devices can then either attach on exposed surface or relocate through water runoff. Massive amounts of such droplets can conduct resource and environmental surveys through integrated humidity, gas, temperature, and pollution sensors. In addition, the droplets can be readily applied through pipettes or syringes. Their manipulation can be achieved through external or internal forces generated by magnetic fields,^16^, ^17^ electrical fields,^18^, ^19^ or chemical reactions,^20^, ^21^ resulting in motion in both dry and wet conditions and capabilities of reaching narrow space that is inaccessible by conventional robots. By combining state‐of‐the‐art flexible electronics, it is feasible for the droplets to achieve customized functions including, but not limited to, energy harvesting, sensing of physical and chemical parameters, and actuation in a self‐sustainable manner.

A conceptual flexible electronic device to integrate with a droplet (Figure 1b and Figure S1, Supporting Information) is 6.5× 6.5 × 0.009 mm^3^ in dimension with a mass of 1.1 mg (Figure S2, Supporting Information). The detailed fabrication processes of this flexible device have been presented in Figure S3 (Supporting Information). The resulting device contains multifunctional components to conduct hydration, temperature, strain, and environmental light monitoring as well as electrochemical sensing to determine biomolecule concentrations. The temperature sensor can also be used reversely as a heater for thermal actuation. In addition, a wireless power scavenger that contains a coil, a rectifier, and a microLED (µLED) has also been included to harvest near‐field radio frequency power. The combination of surface tension and a large surface area to mass ratio of the flexible devices results in free float of devices on the surface of the droplets without submerging (Figure 1c,d), while the flexibility of the devices allows compatible device deformation when the droplets undergo shape shifting. The adhesion forces between water molecules and the flexible devices ensure no interfacial separation between the droplets and the devices even under extreme deformation and rapid motion. Regular liquid media can also be replaced by magnetofluids (e.g., ferrofluids), resulting in controllable droplet motion driven by magnetic force (<0.4N) on superhydrophobic surface (Figure 1e). When a droplet is in dynamic motion, a flexible device placed along the path of the droplet can be picked up by the droplet (Figure 1f), and stay on the top of the droplet due to equilibrium of surface tension force and weight, resulting in synergic motion of both droplet and flexible device.

### 2.2 Deployment of ADs through Artificial Precipitation

**Figure 2:**
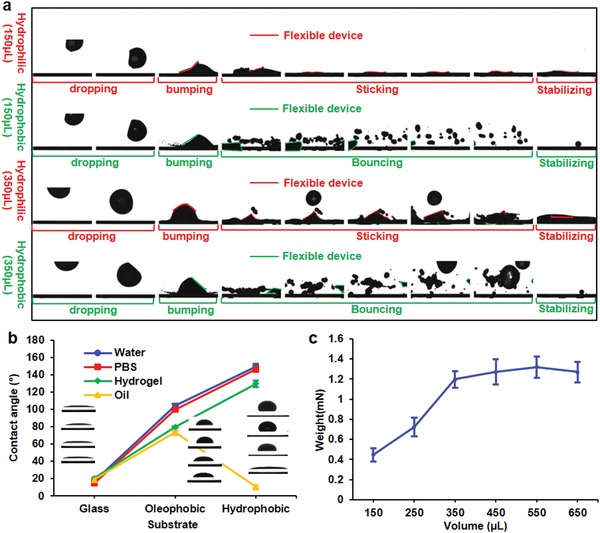
Deployment of active droplets. a) Varied behaviors of droplets when fall onto hydrophobic or hydrophilic surfaces. b) Contact angles of droplets of different composition materials before and after surface treatment of substrates. c) Maximum weights that droplets can carry change with respect to droplet volumes

The behaviors of droplets and integrated flexible devices in response to free‐falling events that simulated situations of large‐scale deployment through rainfall have been investigated. The dynamic motion in a short time period before and after impact onto superhydrophobic and hydrophilic surfaces have been recorded (**Figure** **2**a). The terminal velocity of the droplets before impact was calculated to be 2 m s^−1^, which was on the same scale as the typical terminal velocity of rainfall. The droplets served as viscous dampers that counteracted the impact force applied on the flexible devices. In the case of hydrophilic surfaces, a small volume (150 µL) of droplets resulted in quick water spread (Movie S1, Supporting Information), while a large volume (350 µL) of droplets caused small secondary splash of water. Despite the water splash, the flexible devices had a tendency to stick on the regions near the first impact points and did not follow the secondary splash. On the other hand, the droplets splited into several smaller fragments that keep bouncing on the superhydrophobic surfaces until complete consumption of the potential energy. The flexible device stayed with one of the biggest fragments until bouncing out of the window of observation. In all four experiments, the flexible devices maintained on top of the droplets without direct contact to the rigid surface during the impact processes, demonstrating feasibility to conduct airborne transportation of ADs due to effective protection of the flexible devices from the underneath water droplets.

### 2.3 Characterization of ADs

The complexity to trigger large droplet motions can be overcome by modifying the interfacial energy between droplets and underneath surface. As superhydrophobic surface promotes droplet motions by lowing the interfacial energy, thus, all surfaces for demonstrating droplet motions were, therefore, modified with a kind of nanoparticle spray to yield interfacial nanostructures (Figure S4, Supporting Information) that cause Cassie–Baxter effect due to interfacial air trapping. As a result, the contact angles of different droplet media (pure water, phosphate buffered saline (PBS), hydrogel, and oil) increased from 15.8°, 18.3°, 19.6°, and 11.1°, on untreated glass slides, to ≈154.1° for water‐based media on the superhydrophobic surface, and 71.1° for oil‐based media on the oleophobic surface (Figure 2b). The fraction of the water/solid contact surface area (*f*) has been reduced to 0.051 according to cos θ_*r*_ = *f*(cos θ* + 1) − 1, in which θ* and θ_r_ are the contact angles on the untreated smooth surface and the superhydrophobic surface, respectively.^22^ The changes in contact angles also suggest increased roughness and surface area, making the droplets have greater tendency to move under disturbances and external forces.

The capabilities of different volumes of droplets in carrying weights were characterized using stacked flexible electronic devices with the same dimensions and different numbers of layers. Small droplets (150 µL) can carry a weight of 0.45 mN, while large droplets (>350 µL) withstand relatively stable loading capacities up to 1.32 mN (Figure 2c). In an ideal case, where the resultant forces (due to surface tension) are completely upright, the theoretical maximum device weight is 1.89 mN, which can be calculated by the surface tension force through *Lγ*cosθ, where *L* is the circumference (26 mm) of the device, γ is the surface tension of water (0.073 N m^−1^), and θ is the contact angle (0°). As the circumference is fixed for current device design and the surface tension of the water/air interface is a constant, thus, the maximum carrying weight, which is a counteract force to the surface tension force should eventually reach a fixed value for the same circumference. For small droplets, as the device is comparably large, thus, the device is bended to fit the geometry of the droplet, resulting in increased contact angle and small surface tension force. In addition, the instability caused by disturbance when putting the stacked devices to the droplets further limits the experimental maximum weight to approach the theoretical values.

Based on current device design, the required external pull‐up force to separate the device from the droplet is ≈1.9 mN, which is determined by the weight of the flexible device and the adhesion force (surface tension force) of the water. In the droplet moving situation (Figure 1e), the flexible device is subject to the surface tension force and the inertial force (*ma*). Separation of droplet with the flexible device happens when the inertial force is larger than the surface tension force. As the mass (*m*) of the device is very small (1.1 mg), the acceleration (*a*) has to be larger than 1718 m s^−2^, which is impossible to happen in practical situations. Thus, it is safe to assume that the separation of flexible devices and droplets is unlikely to happen in the normal operation conditions. However, if further increasing the weight of the flexible devices to a maximum value of 1.32 mN while maintaining device floating without submerging (Figure 2c), the separation acceleration reduces to 14 m s^−2^ for large droplets (>350 µL) and 41 m s^−2^ for large droplets (150 µL).

### 2.4 An Electromagnetic Platform for Driving Motion of ADs

**Figure 3:**
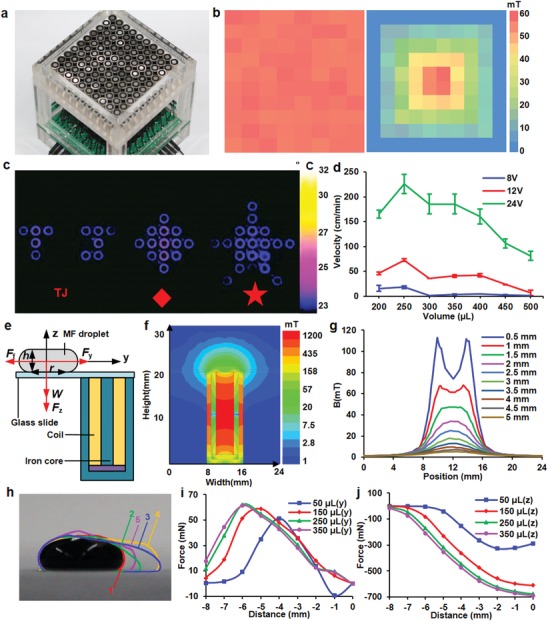
Driving ADs through an electromagnetic platform. a) 10 × 10 arrays of electromagnets driven by a backplane formed by diodes and relays. b) Homogenous and inhomogeneous distribution for the electromagnetic field, defined by applied voltages to an individual electromagnet. c) Thermal images of different patterns generated due to repeated cycling of designated electromagnets. The cycling paths were predefined to form patterns, such as letters, diamonds, and stars. d) Velocities of varied volumes of droplets driven by the electromagnetic platform at different voltages. e) A diagram that shows the structures and components used for simulation. f) Distribution of magnetic field surrounding the electromagnet. g) Magnitude of B field of the electromagnet with varied distances. h) Outlines of shape changes of a droplet when the droplet moved from one electromagnet to the other. All outlines were superposed together to assist visualization and labeled with numbers to indicate the sequence in which the deformation happened. Simulated magnetic forces along i) vertical and j) horizontal directions applied on the moving droplet

The manipulation of droplets was achieved by a platform equipped with 100 electromagnets (**Figure** **3**a) arranged side‐by‐side to form 10 × 10 arrays (10 × 10 cm^2^ in dimension). Each electromagnet can be individually addressed through a rear panel, which contains arrays of switching diodes and relays (Figure S5, Supporting Information). The electromagnets can be driven by a voltage lower than 24 V, allowing generation of a uniform magnetic field with an average intensity of 60 mT and a standard deviation of 0.2 mT. It is also feasible to generate a gradient of the magnetic field by controlling the driving voltage of the arrays (Figure 3b), allowing varied moving velocities of droplets on the platform. The cycling of electromagnets can be preprogramed, resulting in varieties of droplet motion patterns (e.g., letters, diamonds, and stars) defined by the electromagnet arrays and measured as temperature changes within individual electromagnets (Figure 3c). It can be observed that the temperature increase is less than 2 °C after 30 repeated cycles. The increase of temperature is sufficiently small to prevent significant droplet evaporation. The electromagnetic platform is scalable to accommodate the need for droplet manipulation in large areas, and is more sophisticated than other magnetofluidic (MF) driving approaches based on single permanent magnets.

The magnetic force applied through the electromagnetic platform underneath the droplets can induce motions of MF ADs, which contain Fe_3_O_4_ nanoparticles (50 nm in diameter) dispersed in water. The changes in driving voltages of electromagnets offer various moving velocities of the droplets (Figure 3d). An average velocity of 226 cm min^−1^ can be achieved for a droplet (250 µL in volume) under a driving voltage of 24 V. Lowering the driving voltage in half reduced the average velocity to only 72.8 cm min^−1^. At a driving voltage of 8 V, the average velocity reduced further down to 18.1 cm min^−1^. Droplets of other volumes exhibited similar nonlinear relationships between velocities and driving voltages. However, they only achieved smaller average velocities as compared with the one at 250 µL, which was then determined to be the optimum volume to offer the fastest droplet motion. The MF droplets are currently only move on hydrophobic surface and in immiscible liquid. As the droplets spread over the hydrophilic surface, causing large friction force due to increased contact area. Thus, the attraction force supplied by the surrounding electromagnet is not strong enough to overcome the friction force. However, if replacing the electromagnet with permanent magnet to generate larger attraction force. The magnet may cause serval spikes on the droplet. These spikes may eventually separate from the mother droplet and fly over to the magnet, making the motion of the droplet as a whole very difficult.

### 2.5 Simulation of Magnetic Field Generated by Electromagnets and Magnetic Forces on Droplets

Driving the motion of ADs by electromagnetic field is critical to achieve mobility of ADs, and is challenging due to rapid attenuation of B field with increased distances. Simulation of electromagnetic field distribution and electromagnetic forces applied on droplets with varied volumes was conducted using Maxwell. As shown in Figure 3e, the simulated system contains a droplet on a glass slide, which is placed above an electromagnet. According to the simulation results, the B field quickly attenuates from 74.5 mT on the surface of the glass slide directly above the electromagnet to only 5.11 mT in a distance 5 mm above the glass slide (Figure 3f). In addition, significant edge effect can be observed on the surface of the glass slide, as the B field varies from 74.5 mT on the center to 112.2 mT on the edge (Figure 3g). The edge effect gradually vanishes with increased distances from the electromagnet. The nonlinearity of the B field suggests complex magnetic attraction forces applied on the droplets.

Motion of ADs is subjected to combined forces resulting from surface tension, magnetic forces, and friction forces, leading to complex, and nonuniform creeping motions that involve alternating acceleration and deceleration (Movie S2, Supporting Information). We superposed the contours of an AD when it moved from one electromagnet to a neighboring electromagnet in a time sequence (Figure 3h). The results indicate stable receding contact angles (≈158°) and small variation in advancing contact angles (Figure S6, Supporting Information). Assuming that the surface tension and droplet weight did not vary during the motion, the deformation of the ADs was mainly caused by magnetic forces represented by two scalars (*F*_y_ and *F*_z_) along horizontal (y) and vertical (z) directions (Figure 3e). With reduced distances between the droplets and the center of the electromagnet, the simulated *F*_y_ exhibits parabolic changes (Figure 3i), while *F*_z_ shows monotonous increase (Figure 3j). The increased *F*_z_ indicates necessity to reduce friction coefficient of the glass slide by creating superhydrophobic surface to ease droplet motion driven by small *F*_y_.

### 2.6 Motions of ADs Driven by Magnetic Force or Potential Energy

**Figure 4:**
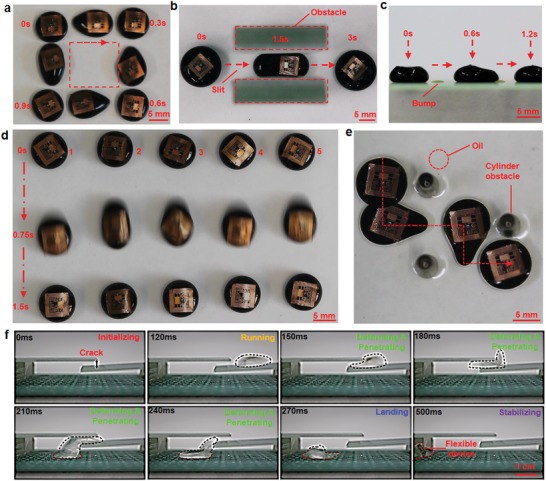
Demonstration of droplet motions. a) A droplet moves in a square‐shaped path. b) A droplet moves through a narrow slit and has reversible shape changes. c) A droplet moves across a surface with small bumps. d) Five droplets move in the same direction in a synchronized manner. e) A droplet moves in oil while dodging obstacles. f) A droplet moves across a gap formed by two overlaid glass slides, and delivers a flexible device to the PCB board underneath it

The platform can drive droplets in well‐controlled manners as demonstrated by several representative motions in both dry and wet environments. First, two‐axial translation movement was demonstrated by driving an MF droplet to move in a 4 × 4 cm^2^ square (**Figure** **4**a and Movie S3, Supporting Information). For locations that are difficult to access, the liquid droplets can pass through narrow slits arranged in vertical (Figure 4b and Movie S4, Supporting Information) and horizontal (Figure S7 and Movie S4, Supporting Information) directions. This results in adaptive deformation within the slits and quick recovery to the original geometry after passing through. In addition, the droplets can tolerate certain levels of surface obstacles. As shown in Figure 4c and Movie S4 (Supporting Information), when small bumps (thickness of 1 mm and diameter of 3 mm) were deliberately created along the path of droplet movement, the droplets overcame the bumps and passed over smoothly. Besides controlling a single droplet, simultaneous control of five droplets that moved side‐by‐side with each other (Figure 4d and Movie S3, Supporting Information) demonstrate the capability of conducting high‐throughput chemical synthesis^23^ by manipulating different droplets on a single platform. Besides moving on dry surfaces, the ADs are also able to swim within a liquid media that is immiscible to the droplet materials. As shown in Figure 4e and Movie S3 (Supporting Information), a water‐based droplet can carry a flexible device and conduct complex zigzag movements to dodge obstacles in silicone oil. This ability is extremely useful in biology where different cell colonies and analytes need to be serially accessed and continuously monitored in multisteps evaluation processes.^24^ When a feedback control mechanism is implemented to reflect the real‐time position of an individual droplet, such a platform can be more intelligent and automatic to perform tasks that involve complicated droplet manipulations, such as combining, separating, asynchronous motions, and precise assembly.

It is possible to use water droplets as a carrier to transport flexible electronic devices to locations that are difficult to access through conventional and superficial layer‐by‐layer assembly approaches. As shown in Figure 4f, two overlapping glass slides act as barriers and form a small gap (5 mm in height) that hinders direct access to the printed circuit board (PCB) circuit underneath it. Adding extra components to the circuit, for status monitoring and circuit modification, is extremely difficult without disassembling the glass slides. However, the ADs (350 µL in volume) were able to pass through the gap while undergoing extreme deformation. The flexible circuit, eventually reached the PCB circuit, and fixed on the circuit through droplet evaporation. The deformation of these ADs and the adaptability of the flexible electronics to its carriers are effective, suggesting potential applications for such ADs in completing complicated tasks in space‐constrained environments that are not feasible for devices and carriers in conventional formats.

### 2.7 Experiments of Flexible Electronic Devices on Droplets

**Figure 5:**
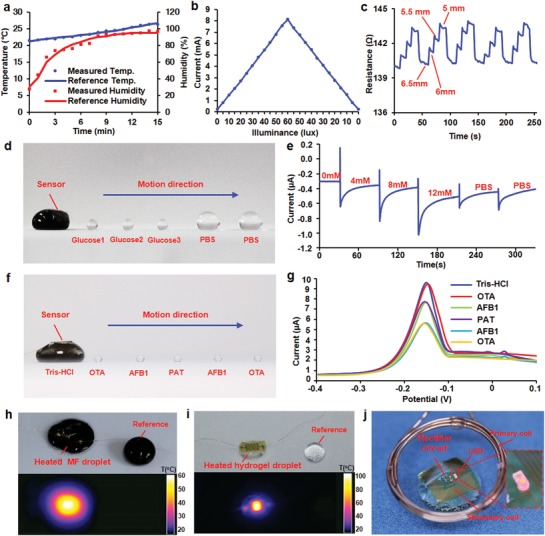
Demonstrations of the functions of ADs. a) Synchronized temperature and humidity variations measured by both an AD and a commercial system. b) Environmental light intensity measured by a phototransistor in an AD. c) Repeated strain changes measured by a CNT strain sensor in an AD in response to changes in the width of the slit where the droplet is located. d) An AD with an electrochemical sensor for glucose concentration measurement. e) The corresponding sensor signal, when a glucose sensitive droplet moves and merges with droplets along its path. f) An AD with an electrochemical sensor for AFB1 identification. g) The corresponding sensor signal when an AFB1‐specific droplet moves and merges with droplets along its path. h) A flexible heater generates heat to evaporate an underlying droplet. i) A flexible heater induces liquid/solid transition of a temperature‐sensitive hydrogel droplet. j) Demonstration of wireless power harvesting with an AD

The functions of flexible electronic devices on droplets have been demonstrated through environmental sensing, glucose concentration determination, radio‐frequency power harvesting, and thermal actuation using discrete sensing and power harvesting elements. A droplet integrated with the conceptual flexible device was placed in a controlled environment simulated by a beaker, while a commercially available device was also in the beaker to provide reference humidity and temperature results (Figure S8, Supporting Information). Both the resistance of the temperature sensor and the impedance of the humidity sensor closely follow the recorded reference values. As the reference temperature changed from 21.2 to 26.7 °C, the resistance of the flexible sensor changed from 28.9 to 29.4 Ω, corresponding to measured temperature changed from 21.5 to 26.4 °C (**Figure** **5**a). Simultaneously, the reference humidity changed from 30% to 95%, when the measured humidity changed from 27.8% to 97.1%. The device was then placed directly underneath a light source that generated varied illuminance. A thin film phototransistor in the flexible device generated varied source/drain currents (*I*_sc_) from 0.2 to 8.1 mA in corresponding to changing light intensities from 0 to 60 lux (Figure 5b). To mimic situations when droplets are passing through slits while simultaneously evaluation of slit width is needed, the strain sensor within the flexible device was used to access droplet deformation, which was directly related to the widths of slits. When the width of a slit, formed by two glass slides, changed from 6.5 to 5 mm, a droplet sandwiched between the two slides was deformed to adapt to the changing widths. This led to increased resistance of the strain sensor by ≈1% for each 0.5 mm compression due to increased bending curvature. The resistance changes exhibited high reversibility with small drifts for less than 0.1 Ω after 5 repeated deformation cycles (Figure 5c).

In addition to physical sensing, the droplet also performed electrochemical sensing demonstrated by glucose concentration monitoring. In a dynamic measurement, an MF AD that originally contained no glucose, merged with three glucose droplets (4, 8, and 12 mmol L^−1^, respectively) and two PBS droplet that scattered along the movement path of the AD (Figure 5d). When the AD successively combined with other droplets, the corresponding signal changed by 0.2 µA when overall glucose concentration changed from 0 to 857 µmol L^−1^. A reverse response was observed when the droplet switched to PBS, leading to a final glucose concentration of 462 µmol L^−1^ (Figure 5e and Movie S5, Supporting Information).

To further demonstrate the application of ADs in mobile chemical sensing, a droplet that contained Aflatoxin B1 (AFB1) aptamers has been used to identify AFB1 from other interfering mycotoxins. When the AD merged with five mycotoxin droplets arranged in a sequence of Ochratoxins A (OTA), AFB1, Patulin (PAT), AFB1 and OTA along the path of the AD (Figure 5f). The *I*/*V* curves of electrochemical sensor only changed in response to AFB1 droplets, the corresponding signal changed by 2 µA for each combination (15 min for incubation), no response was observed when the droplet switched to OTA and PAT (Figure 5g). These results indicated that the AFB1 aptamer‐based electrochemical sensor has a high specificity for AFB1 mycotoxins and could distinguish AFB1 in droplets.

The temperature sensor in the flexible device can also be used as an actuator, which can generate heat to cause phase transition of the underneath droplets. For a regular water droplet, the heater generated a temperature higher than 60 °C (Figure 5h and Movie S6, Supporting Information), completely eliminating the droplet through evaporation. As a result, the position of the flexible device can be fixed at the location where the droplet evaporated. In addition, when temperature sensitive hydrogel (methoxy poly(ethylene glycol)‐*b*‐poly(lactide‐co‐glycolide) was used as the carrier, the heater was able to generate temperatures up to 100 °C (Figure 5i), resulting in reversible phase transition of the hydrogel from a liquid state (transparent) to a solid state (white). Besides of heating, other actuation approaches, such as cooling, vibration, and light emission may be integrated with flexible devices to actively alternate droplet properties that are sensitive to temperature, force, and photonic energy. Finally, a flexible wireless power scavenger was demonstrated on a droplet (Figure 5j). When expose an electromagnetic field with a frequency of 81 kHz, the wireless power scavenger was able to harvest sufficient radio frequency power to light up a LED, suggesting the feasibility of achieving self‐sustainable droplets that operate with remote powering. The demonstration of above functions, including sensing, actuation, and power harvesting, represents promising potentials of such droplets in performing complicate tasks that demand high levels of self‐awareness and machine intelligence. In practical applications, such a multifunctional device can also be made by two separated devices that are stacked together back to back and physically connected through curable polymers, resulting in direct contact with analytes and surrounding environment of a device that has sensing components facing both upward and downward.

## 3 Discussion

The ADs presented here represent innovative hybrid systems that combine the unique properties of liquid droplets with flexible electronics to perform sophisticated tasks, such as sensing, actuation, motion, and power harvesting. The droplets can adaptively and reversibly alter their geometry to accommodate the surrounding environment, while maintaining their functionality through intact flexible electronic devices. The ADs reveal new types of deformable artificial organism that can be miniaturized and organized to complete time‐consuming and labor intensive assignments, such as gene‐expression analysis,^25^, ^26^ chemical synthesis,^27^, ^28^ drug discovery,^29^, ^30^ and microassembly^31^ that demand complicated liquid operations and continuous monitoring. The ADs can also be deployed in large scales to offer collected efforts in monitoring environmental conditions and pollutants, or even charging localized environments. The multiphysics coupling among liquid droplets, flexible electronics, and force fields generates interesting phenomena in mechanics and electronics, and demands further studies to optimize the coupling efficiency as well as functionality.

One of the attractive features of such ADs is their dynamic motion, which is currently driven by either potential energy or magnetic fields. Considering the wide availability of various droplet‐driving mechanisms, it can be foreseen that more driving approaches, such as electrowetting,^32^ acoustic wave,^33^ pressure gradient,^34^ and chemical reaction^21^ may also be feasible to achieve droplet dynamic motion. It can also be expected that the ADs may eventually become more intelligent to allow low‐energy and self‐sustainable operation in preprogramable or even self‐aware manners, resulting in intelligent droplets that can work in different territories, such as land, water, and air. Further investigations can be conducted to achieve a more sophisticated platform that will allow synchronous or asynchronous manipulation of multiple droplets by introducing real‐time position sensing and feedback control. A platform may also be constructed by flexible electronics technology to yield low profile and foldable configuration that can be portable and easily deployed.

## 4 Experimental Section

*Design of the Flexible Electronic Device*: The conceptual flexible device contained physical sensors, chemical sensors, and an energy scavenger, all of which served for demonstrating the potential appearance of active droplets in future applications. The physical sensors included temperature sensors, humidity sensors, strain sensors, and light intensity sensors. The chemical sensors used electrochemical detection approaches to monitor glucose concentrations. The energy scavenger harvested environmental radio‐frequency energy and powered a LED through a half wave rectification circuit. The temperature sensor was based on a thin layer (800 nm) of copper in a meander structure (600 × 500 µm^2^ in dimension) with an original resistance of ≈28 Ω. The humidity sensor contained a pair of capacitive circular electrodes that included an internal circular plate (240 µm in diameter) and an external ring (500 µm in inner diameter and 800 µm in outer diameter). The impedance of the circular electrodes changed with environmental humidity. Two strain sensors arranged perpendicular to each other were based on two pairs of single wall carbon nanotube (SWCNT) electrodes, which were designed to have a 10:1 length‐to‐width ratio that ensured uniaxial response to external strain. The light intensity sensor was made of a thin film phototransistor (690 × 690 µm^2^ in dimension), which was thinned down from an original thickness of 200 µm to a thickness of less than 30 µm. The source and drain electrodes in the phototransistor were connected with flexible interconnects, while the gate electrode was floating. The chemical sensor had a three‐electrode configuration with an overall dimension of 0.5 mm^2^. The reference electrode (RE) was deposited with an Ag/AgCl bilayer. The working electrode (WE) can be modified with glucose oxide enzyme to conduct glucose detection. The power scavenger contained a Cu coil that was connected with a half wave reification circuit formed by a diode, a parallel capacitor, and a µLED.

*Fabrication of the Flexible Electronic Device*: The fabrication processes of the flexible electronic device are shown in details in Figure S3 (Supporting Information). Fabrication of the flexible electronic device began with spin coating a polydimethylsiloxane (PDMS) layer on a glass substrate to form an adhesive layer. A copper membrane (3 µm in thickness) coated with a layer of polyimide (PI) (1 µm in thickness) was then attached on the PDMS layer followed by depositing a Ti/Au (5/25 nm in thickness) bilayer. A silver (Ag) layer was then electroplated onto a defined region to form the RE for the glucose sensor, followed by immersing the RE into a FeCl_3_ solution (50 × 10^−3^ m) for 50 s to obtain a thin layer of AgCl on the Ag layer. Patterns of the glucose sensor, the humidity sensor, the interconnects, the bottom capacitive plate, and the wireless coil were all obtained through wet etching of Cu/Ti/Au multiplayers. A second layer of PI was then spin‐coated and etched to define vias and contact pads through the PI layer. A thinned Schottky diode and a phototransistor were then transferred printed and electrically connected to the corresponding contact pads through silver epoxy. A diluted SU‐8 photoresist was spin‐coated as a thin adhesive layer, followed by µLED transfer‐printing and SU‐8 curing at 95 °C for 3 min. A second layer of SU‐8 was then spin‐coated to cover the entire substrate, followed by patterning SU‐8 layers to only encapsulate the diode, the phototransistor, and the µLED, while exposing corresponding pads for subsequent interconnection. A Ti/Cu bilayer (5/800 nm in thickness) was formed through sputtering, and was patterned to connect the diode, the phototransistor, and the µLED to the bottom interconnects. In addition, the patterned Ti/Cu bilayer also formed the top capacitive plate and the temperature sensor. A SWCNT solution (TNWDSR, Chengdu Organic Chemicals Co., Ltd.) was spin‐coated on the region for strain sensors, and was patterned through oxygen plasma etching to form L type perpendicular sensor strips. A final layer of PI was at last deposited to insulate the entire devices, and was patterned to expose the electrode for the glucose sensor, the humidity sensor, and connection pads for connecting with external testing equipment. The flexible electronic device can be readily picked up from the PDMS layer by a water‐soluble tape, which can be dissolved later to release the device.

*Modification of WEs for the Flexible Electrochemical Sensors in ADs*: The WE of the flexible glucose sensor was modified by 1 µL of a *N*,*N*‐dimethylformamide (DMF) droplet dispersed with graphene and multiwall carbon nanotubes (mixed with a mass ratio of 1:1). 1 µL of glucose oxidase solution (80 mg mL^−1^, 9001‐37‐0, Dalian Meilun Biotechnology Co., Ltd.) was then drop‐casted to the modified WE and dried in a refrigerator at 4 °C for 30 min. Finally, Nafion (5 wt% in lower aliphatic alcohols and water, 274 704, Adamas Pharmaceuticals, Inc.) was drop‐casted to seal the WE and dried at room temperature for 20 min. The WE of the flexible AFB1 sensor was also modified by 1 µL of a DMF droplet dispersed with graphene and multiwall carbon nanotubes and dried at room temperature for 30 min. Gold nanoparticles (GNPs) were then obtained on the WE by electrodeposition at the potential 0.1 V for 15 s using electrochemical Workstation. Next, 3 µL of the AFB1 aptamer (5′‐GT TGG GCA CGT GTT GTC TCT CTG TGT CTC GTG CCC TTC GCT AGG CCC‐SH‐3′, Sangon Biotech (Shanghai) Co., Ltd.) solution (100 nmol L^−1^) was drop‐casted to the WE and dried at 37 °C for 2 h. Finally, the sensor was successively rinsed by Tris‐HCl buffer (10 mmol L^−1^, PH = 7.0, MKM Biotech (Tianjin) Co., Ltd.) and water, and dried at room temperature.

*Preparation of MF Droplets*: Magnetofluid was obtained by mixing water, polyvinylpyrrolidone (PVP, MW: 10000, 89088C, Adamas Pharmaceuticals, Inc.) and Fe_3_O_4_ nanoparticles (50 nm in diameter) (DK401, Beijing Deke Daojin Science and Technology Co., Ltd.) with a weight ratio of 4:1:1 by sonication for 15 min.

*Construction and Characterization of a Multichannel Electromagnet Platform*: The multichannel electromagnet platform contained 100 electromagnets (YHN‐P08/20, Pingheng Electromechanical Co.) arranged to 10 × 10 arrays and driven by a PCB controlling circuit that contained 100 diodes each of which corresponded to an electromagnet. The diodes in the PCB circuit allowed current to flow in a single direction to prevent crosstalk between electromagnets. The electromagnets were fixed into an acrylic frame that was then covered with a superhydrophobic glass slide. A LabVIEW program was developed to allow access to individual electromagnet through a NI DAQ controller (USB‐6501, National Instruments) that controlled 10 row‐selection relays and 10 column‐selection relays.

To verify the uniformity of the magnetic field, the LabVIEW program was set to drive each electromagnet for 3 s under a driving voltage of 24 V. A Gauss meter (CH‐1800, CH‐Hall Electronic Devices, Inc.) scanned across the working electromagnet to measure the magnetic field strength. To demonstrate capability to generate different motion paths, a LabVIEW program was preprogrammed to drive electromagnets in defined sequences to form patterns, such as letters, a square box, and a pentagon. For each patterns shown in Figure 3d, each electromagnet was turned on for 300 ms before switching to the next. The temperature on the electromagnets was then measured by a thermal camera (Fotric 111, Fortric Inc.) after 30 repeated cycles.

The simulation of the electromagnet platform was conducted using Maxwell software (ANSYS, Inc.). Two models have been constructed to access the distribution of magnetic field surrounding a working electromagnet and magnetic forces applied on MF droplets. The electromagnet was constructed by following the dimensions provided by the manufacture datasheet. The electromagnet contained copper coils wrapped around an iron core and sealed inside a cylindrical iron enclosure. The coils in the electromagnet were considered as stranded bundles with an overall current flow of 318A (numbers of turn × current in each turn). The MF droplets were treated as circular disks with rounded side walls (Figure 3e). The radii (*r*) of the circular contact area of the MF droplets on the glass slide and height (*h*) of the MF droplets were estimated from the experimental measurements. The resulting volumes from 50 to 350 µL can be determined by *r* and *h* using the following equation

(1) $$2\pi h^{2}r+4/3r^{3}+2h\left(r\sqrt{h^{2}-r^{2}}+h^{2}\mathrm{actan}\left(r/\sqrt{h^{2}-r^{2}}\right)\right)$$

*Construction of the Superhydrophobic*/*Olephobic Glass Slide Substrate*: The superhydrophobic surface and olephobic surfaces were achieved by coating glass slides with a commercially available superhydrophobic coating agent (NeverWet, NeverWet LLC.) and an oleophobic coating agent (K2S, PAIQI‐NANO, Inc.). To obtain superhydrophobic surface, the base coat and the top coat offered by the manufacturer were sprayed on the glass surface and dried at room temperature for 30 min. To obtain the olephobic surface, the K2S agent was spin‐coated on a glass substrate and then dried at 120 °C for 10 min.

*Experiment of Free Falling of Water Droplets*: During the free falling experiment, a stage lifted the droplets to a height of 1 m. Either a superhydrophobic or a hydrophilic slide was placed directly underneath the stage. A pipette was used to blow the droplets out of the stage with near zero initial velocity on the vertical direction. The moment when droplets impacted onto the underneath glass slides was recorded using a high‐speed camera (Ximea MQ013MG‐ON, Ximea GmbH) at a frame rate of 500 fps. The images in Figure 2a and Movie S1 (Supporting Information) were taken when the stage was 20 cm above the glass slides. Experiments using other heights were also conducted with similar results. However, due to difficulty in precisely controlling the impact regions to fall into the field of view of the high‐speed camera, only results with 20 cm distance were presented to offer better visualization of the entire impact processes.

*Characterization of Flexible Electronic Devices*: A controlled environment was created in a beaker on a hot plate. A droplet (250 µL) with integrated temperature and humidity sensors was placed in the beaker on a superhydrophobic glass slide. A commercially available temperature and humidity meter (YHZ‐90450, Yuhuaze, Inc.) was used to provide reference humidity and temperature results. The beaker was preloaded with 300 mL of deionized water, and was sealed with parafilm to prevent water vapor from escaping. The temperature sensor was measured by a source meter (2400, Keithley Instruments, Inc.) and the humidity sensor was measured by a LCR meter (E4980A, Agilent Technologies, Inc.). In the strain sensing experiment, a droplet with a flexible strain sensor was compressed by two pieces of superhydrophobic glass slides, and the resistance changes of the strain sensor was recorded by a multimeter (2002, Keithley Instruments, Inc.). The distance between the glass slides was originally fixed at 6.5 mm, which was then reduced to 5 mm by three compression processes with a step of 0.5 mm. The two glass slides were then returned to the original position. Such a droplet compression and recovery process was repeated for 5 times. To verify the function of light intensity sensing, a droplet (250 µL) with an integrated phototransistor was placed on a glass slide underneath an adjustable LED lamp and connected with a source meter (2400, Keithley Instruments, Inc.). A commercial illuminometer (TES1335, TES Electrical Electronic Corp.) was used to provide reference light intensity results.

In the glucose concentration detection experiment, a PBS MF droplet (250 µL), three glucose droplets (10 µL), and two PBS droplets (120 µL) were placed along the movement path of the AD. The MF droplet combined sequentially with the rest of the droplets, and the response of the glucose sensor was measured by an electrochemical workstation (CHI600E, CH Instruments, Inc.).

In the AFB1 identification experiment, a Tris‐HCl magnetofluidic droplet (250 µL), two AFB1 droplets (2.5 µL, 100 ng mL^−1^), two OTA droplets (2.5 µL, 100 ng mL^−1^), and a PAT droplet (2.5 µL, 100 ng mL^−1^) were distributed along the movement path of the AD. The MF droplet combined sequentially with the rest of the droplets, and the response of the AFB1 identification was measured by the electrochemical workstation.

## Conflict of Interest

The authors declare no conflict of interest.

## Supporting information

Supplementary

Supplementary

Supplementary

Supplementary

Supplementary

Supplementary

Supplementary
