# Supplementary material for: Droplets as Carriers for Flexible Electronic Devices
Source: Adv Sci (Weinh). 2019 Oct 30;6(24):1901862. doi: 10.1002/advs.201901862 (PMC6918117; doi:10.1002/advs.201901862)
Supplement: Supplementary file 1 — Supplementary [file ADVS-6-1901862-s001.pdf]

Copyright WILEY-VCH Verlag GmbH & Co. KGaA, 69469 Weinheim, Germany, 2019.

ADVANCED SCIENCE
Open Access

Supporting Information

for *Adv. Sci.,* DOI: 10.1002/advs.201901862

Droplets as Carriers for Flexible Electronic Devices

*Mingxing Zhou, Ziyue Wu, Yicong Zhao, Qing Yang, Wei Ling, Ya Li, Hang Xu, Cheng Wang, and Xian Huang**

Copyright WILEY-VCH Verlag GmbH & Co. KGaA, 69469 Weinheim, Germany, 2019.

Supporting Information

Droplets as carriers for flexible electronic devices

*Mingxing Zhou, Ziyue Wu, Yicong Zhao, Qing Yang, Wei Lin, Ya Li, Hang Xu, Cheng Wang, Xian Huang**

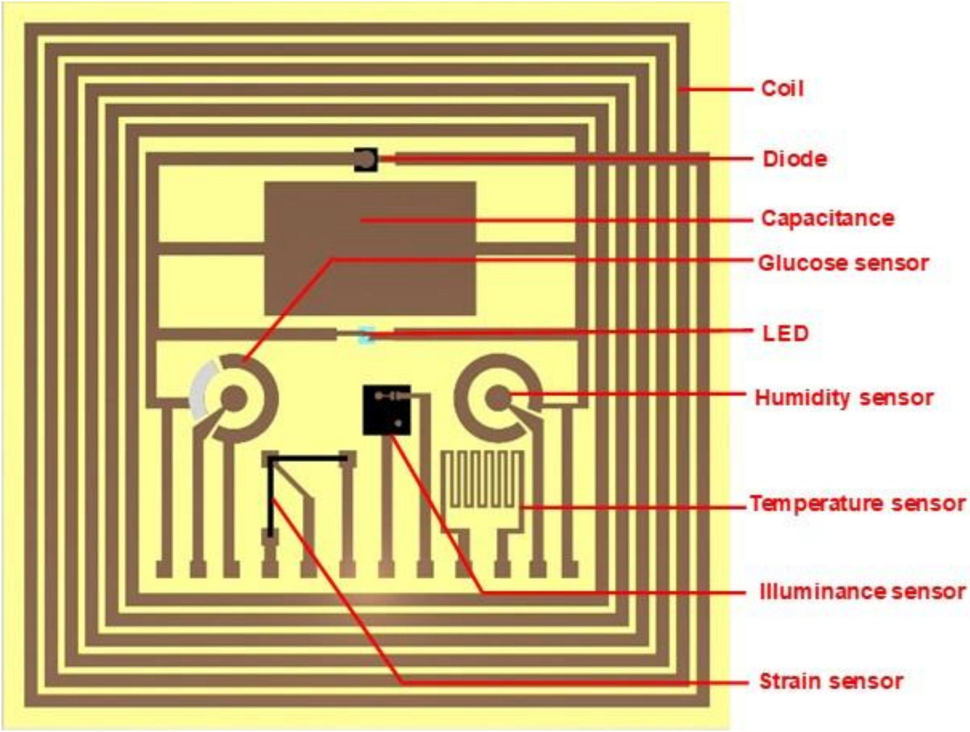

Figure S1. A diagram of the conceptual flexible device.

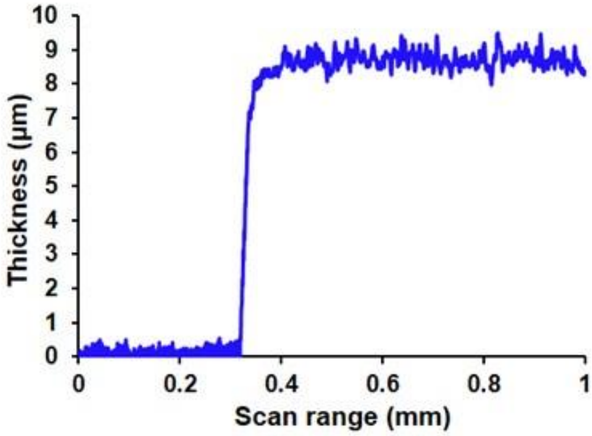

Figure S2. Thickness of a flexible electronics device.

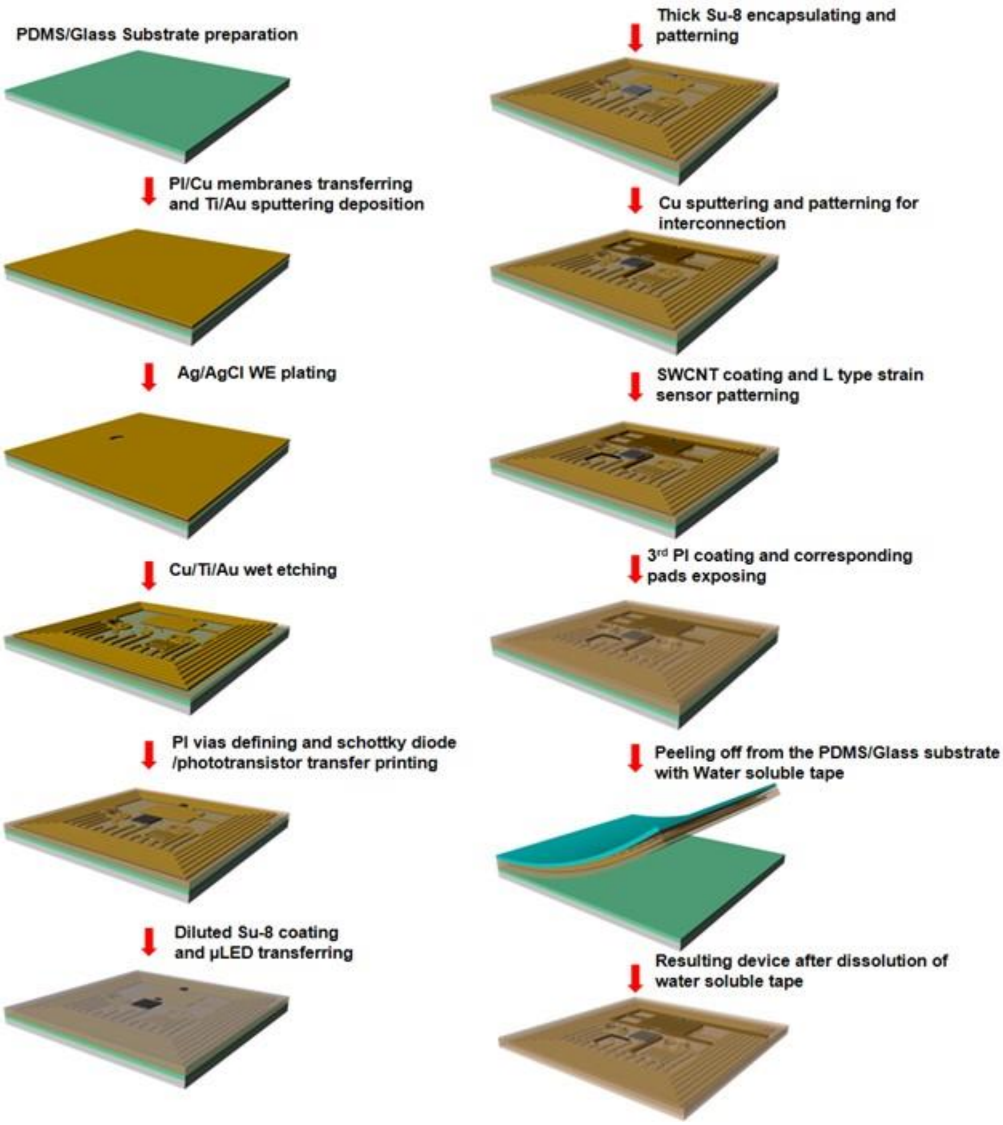

Figure S3. A fabrication process of the flexible device for active droplets.

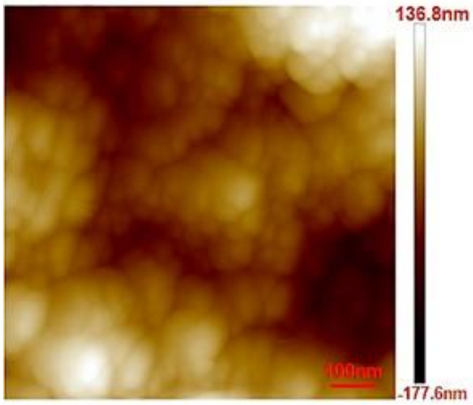

Figure S4. An atomic force microscopic image of nanostructures on the superydrophobic surface.

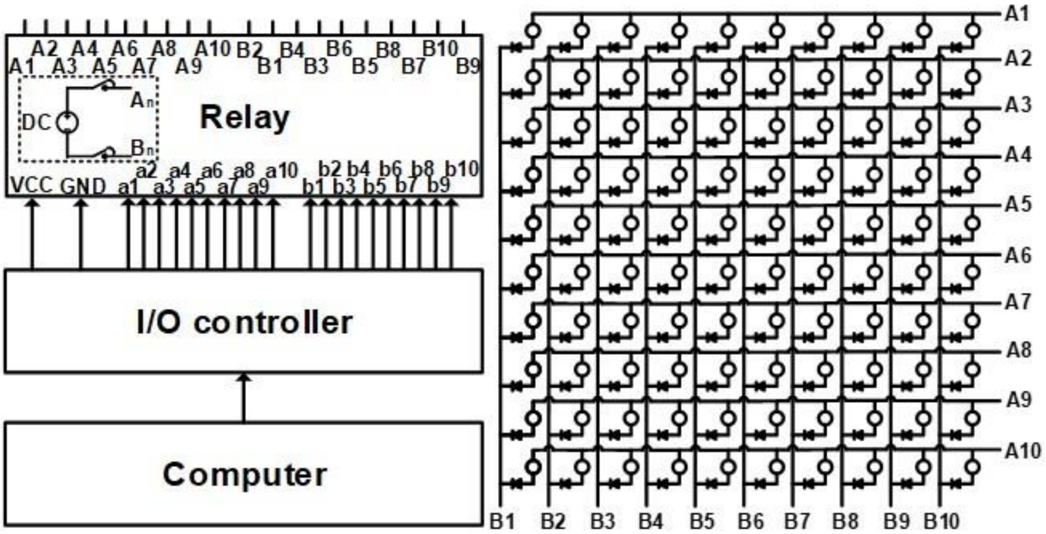

Figure S5. A schematic of the driving circuit for the electromagnetic platform.

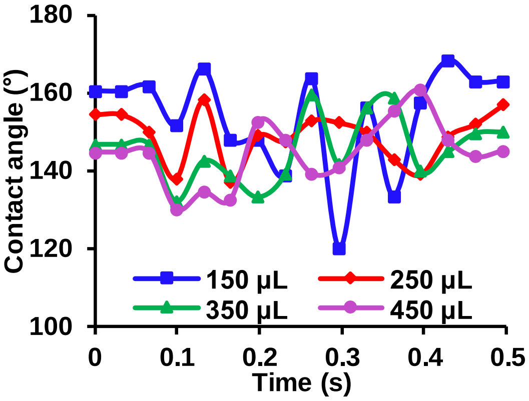

Figure S6. Advancing contact angles of four droplets with varied volumes during a motion process from one electromagnet to the neighboring electromagnet.

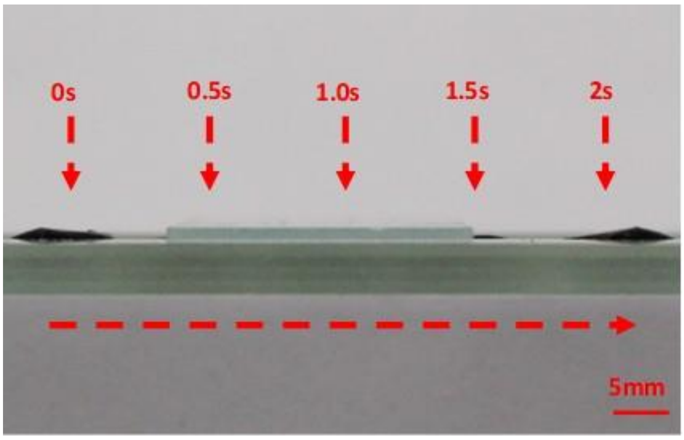

Figure S7. Demonstration of droplet motion. A droplet moves through a narrow horizontal channel and performs reversible shape change.

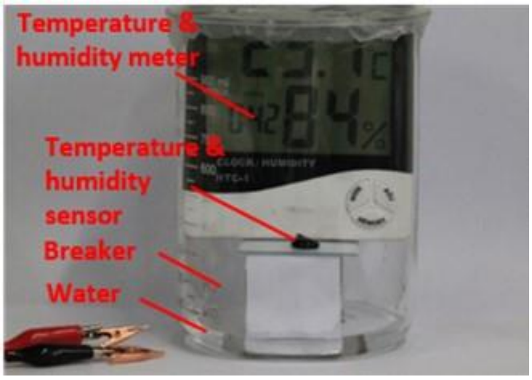

Figure S8. An experimental setup to monitor changes in humidity and temperature.
